# Use of in vitro bone models to screen for altered bone metabolism, osteopathies, and fracture healing: challenges of complex models

**DOI:** 10.1007/s00204-020-02906-z

**Published:** 2020-09-10

**Authors:** Sabrina Ehnert, Helen Rinderknecht, Romina H. Aspera-Werz, Victor Häussling, Andreas K. Nussler

**Affiliations:** Siegfried Weller Research Institute at the BG Trauma Center Tübingen, Department of Trauma and Reconstructive Surgery, University of Tübingen, Tübingen, Germany

**Keywords:** Ex vivo bone cultures, Osteoblast/osteocyte, Osteoclast, Endothelial cells, Co-culture, 2D/3D

## Abstract

Approx. every third hospitalized patient in Europe suffers from musculoskeletal injuries or diseases. Up to 20% of these patients need costly surgical revisions after delayed or impaired fracture healing. Reasons for this are the severity of the trauma, individual factors, e.g, the patients’ age, individual lifestyle, chronic diseases, medication, and, over 70 diseases that negatively affect the bone quality. To investigate the various disease constellations and/or develop new treatment strategies, many in vivo, ex vivo, and in vitro models can be applied. Analyzing these various models more closely, it is obvious that many of them have limits and/or restrictions. Undoubtedly, in vivo models most completely represent the biological situation. Besides possible species-specific differences, ethical concerns may question the use of in vivo models especially for large screening approaches. Challenging whether ex vivo or in vitro bone models can be used as an adequate replacement for such screenings, we here summarize the advantages and challenges of frequently used ex vivo and in vitro bone models to study disturbed bone metabolism and fracture healing. Using own examples, we discuss the common challenge of cell-specific normalization of data obtained from more complex in vitro models as one example of the analytical limits which lower the full potential of these complex model systems.

## Introduction

### Bones of the skeleton are more than the supporting framework for the human body

Based on recent statistical investigations, approx. every third hospitalized patient in Europe suffers from musculoskeletal injuries or diseases. Their treatment makes up to 15% of all therapeutic costs (Eurostat), of which most costs being caused by surgical revisions after delayed or impaired fracture healing. A large British study with almost 3000 patients showed delayed or impaired fracture healing in 20% of these patients (Hernandez et al. 2012). Besides the type and severity of the trauma, individual factors including the patients’ age and individual lifestyle (e.g., reduced physical activity, imbalanced diet, alcohol, or cigarette consumption), as well as emerging chronic diseases and their medication strongly affected fracture risk and fracture healing (Hernandez et al. 2012; Ihle et al. 2017; Schlundt et al. 2018; Sheu and Diamond 2016). Indeed, today, over 70 diseases and health conditions are known that negatively affect bone quality and, thus, increase fracture risk. The increasing prevalence and incidence of such disease-associated changes in bone quality favored the establishment of the terms systemic bone disease and secondary osteoporosis. Facing constantly increasing life expectancy and ageing society, systemic bone diseases and secondary osteoporosis gain more and more relevance. For example, in a representative German level 1 trauma center, 80.5% of all patients suffer from one or more chronic diseases requiring medication (Ø 4.3 drugs per patient): approx. 13% are diabetics (Pscherer et al. 2016b, 2015), 22% are at risk for malnutrition (Ihle et al. 2017), 15% drink alcohol on a daily base, and 42% are smokers (Ehnert et al. 2019a). These patients are above-average in developing post-surgical complications, e.g., delayed wound or fracture healing, which, in turn, results in significantly prolonged hospital stays (Wintermeyer et al. 2018), and often aggravates the underlying disease.

Diabetes mellitus (DM) may serve as good example, representing the metabolic disease with the highest prevalence and incidence in developed countries (Hopps and Caimi 2011). Due to the classical DM-dependent complications, e.g., hypoglycemic or hyperglycemic episodes, impaired vision, or polyneuropathies diabetics have a higher frequency of falls. Due to structural changes of bones [type 1 DM: osteopenia and osteoporosis/type 2 DM: increased bone mineral density (Blakytny et al. 2011; Hofbauer et al. 2016; La Fontaine et al. 2011)], fracture rate is significantly higher in diabetics than in metabolically healthy controls (Hamann et al. 2012; Hofbauer et al. 2007), and seems at least partly to be influenced by the medication obtained (Hidayat et al. 2019; Kalaitzoglou et al. 2019; Kheniser et al. 2018; Pscherer et al. 2016a). For example, diabetics treated with sulfonylurea are commonly associated with a higher risk of falls and fractures (Adil et al. 2017; Lapane et al. 2015; Rajpathak et al. 2015). Similarly, an increased fracture risk has been reported in diabetics receiving glitazones and sodium-glucose transport protein 2 inhibitors for blood glucose control (Lim et al. 2017; Mori et al. 2017; Soroceanu et al. 2004; Watts et al. 2016). The first-line medication for type 2 diabetes is Metformin, which is not associated with an increased fracture risk (Hegazy 2015). Interestingly, studies suggest that incretin-based drugs, e.g., glucagon-like peptide 1 receptor agonists and Dipeptidyl-peptidase 4 inhibitors, can even favor bone health (Nuche-Berenguer et al. 2009; Yang et al. 2017). These data are retrieved from databanks reporting the incidence of fractures. Although this can give valuable advice when choosing a treatment, one cannot conclude on the underlying mechanisms. For obtaining such information, advanced model systems are required.

The example on DM shows how a disease can disturb the balanced interplay between the bone resident cells, controlling bone formation and resorption, either directly or indirectly by its medication. The resulting changes in bone quality may increase the risk for fractures. In case of a fracture, possible disease-related alterations in the local blood vessels (micro- and macro-angiopathies) and inflammation in the surrounding soft tissue, often negatively affect the fracture healing (El-Ganainy and Elgeidi 2010; Kline et al. 2009; Mazziotti et al. 2011; Mehta et al. 2010; Retzepi and Donos 2010; Robinson et al. 2009; Wukich et al. 2011). Therefore, models investigating fracture healing should also address points, e.g., inflammation and vascularization.

There is first evidence that the disease-dependent alterations in the bone also affect other organs within the human body. Researchers in Colorado (USA) proved that production of insulin by β-cells, and thus regulation of blood sugar levels, is tightly controlled by osteocalcin produced by bone forming osteoblasts (Hwang et al. 2011; Kidder et al. 2009; Villafan-Bernal et al. 2011). Hyperglycemic episodes and compensatory formation of insulin suppress maturation of osteoblasts, by stimulating the production, release, and activation of TGF-β (transforming growth factor beta) by osteoblasts, osteoclasts (Ehnert et al. 2010; Ehnert et al. 2017; Freude et al. 2012), and immune cells. Serum levels of active TGF-β are chronically elevated in diabetics, which may act immune-modulatory and pro-fibrotic throughout the entire body (Ehnert et al. 2015; Pscherer et al. 2013). Normally produced to fight excessive inflammation, chronically elevated TGF-β levels in diabetics may suppress acute immune responses and thus favor infections in these patients. Furthermore, TGF-β as a key driver in scar formation is known to favor fibrosis and cirrhosis in various tissues, including kidney, liver, heart, and lung (Fabregat and Caballero-Diaz 2018; Lichtman et al. 2016; Meng et al. 2016; Yue et al. 2017). Thus, DM represents a prime example for the underestimated role of the bones in the human body.

### Cellular interactions in bones

**Fig. 1:**
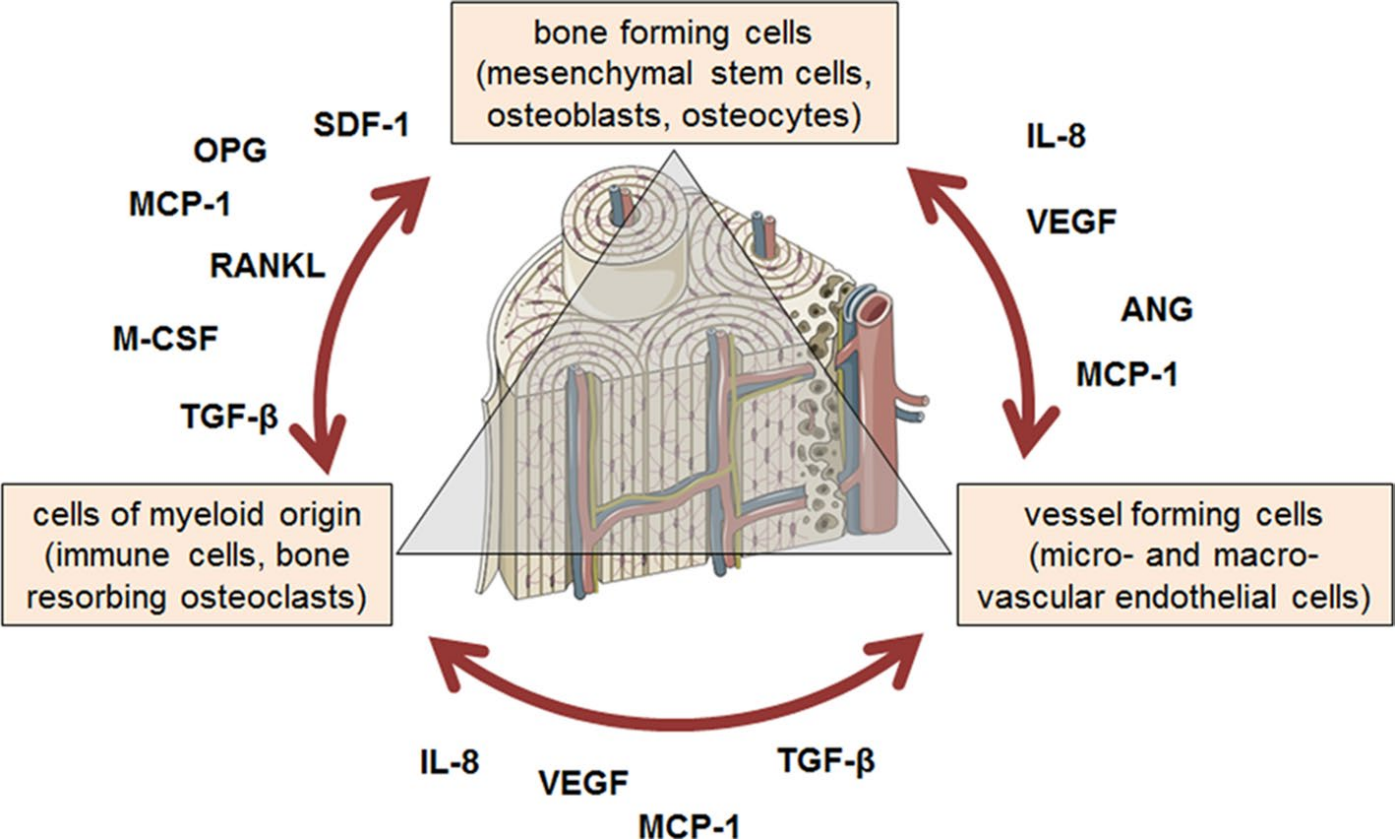
Overview of the different cell types within the bone. Cells resident in the bone communicate with each other by direct cell–cell and cell–matrix interactions, as well as via secreted factors. Representatives of these factors are given in the figure: ANG (angiogenin), IL-8 (interleukin 8), VEGF (vascular endothelial growth factor), MCP-1 (monocyte chemoattractant protein), OPG (osteoprotegerin), RANKL (receptor activator for nuclear factor kappa B ligand), TGF-β (transforming growth factor beta), and SDF-1 (stromal cell-derived factor-1) Bone sketch was obtained from https://smart.servier.com/

Facing new regulations on the re-evaluation and licensing of drugs and medical devices, it should be mandatory to examine the effect of drugs, especially that for sustainable medication, on the bone health. In the human body, bones of the skeleton constantly adapt to the stress exposed. New bone structures are formed by cells of the osteogenic lineage, e.g., mesenchymal stem cells (MSCs), osteoblasts, and osteocytes. Inferior or damaged bone matrix is resorbed by osteoclasts, derived from the hematopoietic lineage. In the bone marrow, new blood cells are produced, which provide bone cells with required oxygen and nutrients via a system of blood vessels (Buckwalter et al. 1996; Florencio-Silva et al. 2015). Therefore, healthy bone function requires an orchestrated interplay between vessel forming endothelial cells, blood cells, bone forming, and resorbing cells, that are often mediated via paracrine and systemic mediators. Yet, little is known about the role of the bone marrow-resident adipocytes (Horowitz et al. 2017). In case of a fracture, additional factors gain importance, e.g., the invading immune cells which represent main drivers for successful bone healing (Kolar et al. 2010; Ma et al. 2019; Pfeiffenberger et al. 2019). Therefore, depending on the intended research interest, in vitro or ex vivo model systems have to display the complex interactions of many different cell types (Fig. 1).

## Models to investigate bone quality and function

### In vivo models

So far, this interplay between many different cell types can only be adequately displayed in vivo in animal models, as the entire composition of cells within the body is given. Providing the natural blood supply, cell–cell and cell–matrix interactions in vivo models remain the gold standard to investigate bone metabolism, systemic bone diseases, or fracture healing (Haffner-Luntzer et al. 2019; Holstein et al. 2009; Simpson and Murray 2015). When the model is based on smaller animals, e.g., mice or rat, the great variety of inbred stains with specific gene over-expression or knock-out can be utilized to investigate specific mechanisms. When fracture healing is investigated the smaller animal models have the advantage, that bones can be broken with a defined force, resulting in a relatively reproducible fracture that represents the pathology of a trauma (Haffner-Luntzer et al. 2019). Despite these advantages, the small anatomy represents a challenge when it comes to fixing the fractured bones (Holstein et al. 2009). Fixation is normally not needed when healing of bone defects with a defined size, which can be drilled into the bones, is investigated. These models show some popularity due to their high reproducibility (Gomes and Fernandes 2011; Harris et al. 2013; Vajgel et al. 2014). With increasing size of the animals, fractures or bone defects may preferably be generated with saws or drills, providing a high degree of reproducibility. Due to the larger size of the bones for example in pigs, dogs, or sheep, the fixation of the fractured bones is quite representable to the human situation. Therefore, larger animals are preferably used to test orthopedic implants or biomaterials (Haffner-Luntzer et al. 2019). Additionally, in some of these models, primary (postmenopausal) osteoporosis can be induced by ovariectomy in female animals (Haffner-Luntzer et al. 2019). This brief overview shows the advantages and great variety of existing in vivo models when it comes to investigating bone metabolism, systemic bone diseases, or fracture healing. However, some limitations remain to be addressed:

- There exist partly huge species-dependent differences in bone metabolism (Aerssens et al. 1998);
- The use of inbred rodent strains cannot display the great inter-individual differences observed in humans;
- Due to strictly controlled housing conditions, animals are not exposed to the same environmental influences as patients, especially in case of a disease;
- Based on the altered posture (tetrapod motion vs upright walk), mechanical strain in bones often differs between animals and humans.
- A considerable amount of animals is needed to obtain representative results.

Taking up the example of DM again, there exist a great variety of animal models, in which DM is induced either genetically (for example Lep^ob/ob^, LepR^db/db^, TallyHo/JngJ, KK, NZO or MKR mice and ZDF, OLETF, or GK rats) or by a specific diet (for example by high caloric and fatty diet) (King 2012; Rees and Alcolado 2005). Focusing on rodent models for type 2 diabetes, bone mineral density often suggests the development of osteopenia and osteoporosis. This is contrary to the human situation, where type 2 diabetics often leads to higher bone mineral density than age- and gender-adjusted controls (Kawashima et al. 2009; Rendina-Ruedy et al. 2016; Rosen and Bouxsein 2006; Won et al. 2011).

Besides associated ethical concerns, this example shows that animal models are only of limited use to investigate metabolic bone diseases or the influence of drugs on the bone. This example underlines the absolute need for models adequately representing the human bone disease.

### Ex vivo bone cultures

**Fig. 2:**
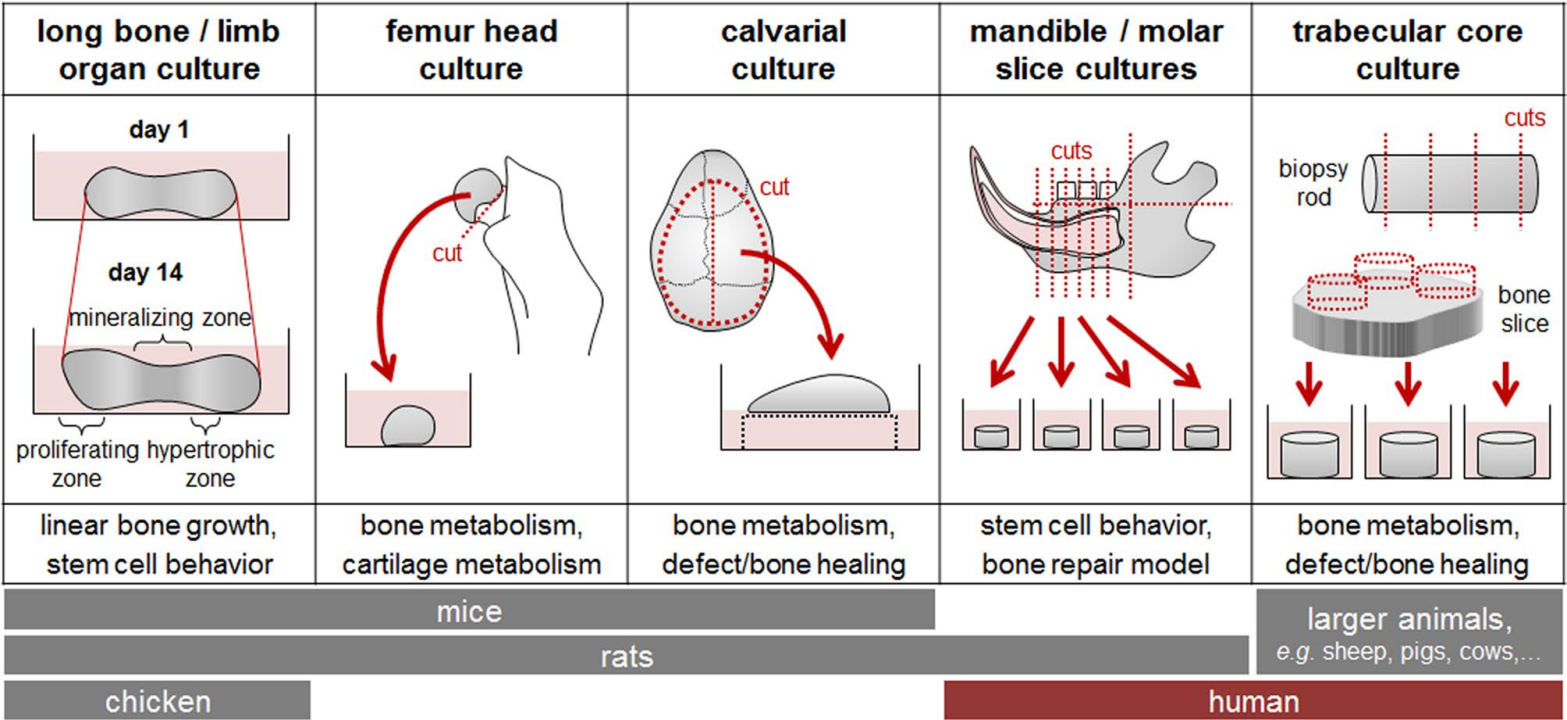
Overview of different available *ex vivo* bone models

Culture models closest to the in vivo situation are the so-called ex vivo organ cultures. Depending on the research interest, different ex vivo bone cultures exist (Fig. 2).

Stem cell behavior during linear bone growth and hypertrophic ossification is best observed in the so-called long bone or limb organ cultures. In the literature, different long bone organ cultures are described (Abubakar et al. 2019; Houston et al. 2016; Muzic et al. 2013; Paradis et al. 2019; Parivar et al. 2006; Proffit and Ackerman 1964; Smith et al. 2013, 2015; Uribe and Rosello-Diez 2019). Already in the 1960s, ex vivo long bone cultures have been described (Proffit and Ackerman 1964): proximal phalanges, metacarpal, and metatarsal bones were dissected from the paws of young rats and kept several days in culture, during which the bones grew and mineralized (Abubakar et al. 2019; Proffit and Ackerman 1964). This method was also described for long bones of mice (Houston et al. 2016; Kunimoto et al. 2016; Okubo et al. 2015, 2013; Uribe and Rosello-Diez 2019) or chicken (Smith et al. 2015). Based on the study from Abubakar et al, approx. 75% of ex vivo bone growth studies are performed using ex vivo long bone cultures (Abubakar et al. 2016). By culturing an explanted bone, the complete cellular composition of the intact organ is provided, representing closely the in vivo situation. This is a great advantage, when bone growth or, to a certain degree, bone metabolism is investigated. Preserving the surrounding soft tissue, during the so-called limb bud cultures promised even better representation of the in vivo situation (Muzic et al. 2013; Paradis et al. 2019; Parivar et al. 2006; Smith et al. 2013). As a fracture model, a limiting factor will be the lack of the vascular system, which is required for the formation of the fracture hematoma (Kolar et al. 2010).

Murine or rat femur head and calvarial cultures (Batushansky et al. 2019; Garrett 2003; Madsen et al. 2011; Mohammad et al. 2008; Sathi et al. 2015) comprise approx. 16% of ex vivo bone growth studies (Abubakar et al. 2016). However, these cultures can also be used for investigating bone and cartilage metabolism or bone defect healing. When co-cultured with other cells, *e.g.,* cancer cells or immune cells, even for investigating bone metastases (Choudhary et al. 2018; Curtin et al. 2012; Marino et al. 2019; Salamanna et al. 2016; Salih 2019) or inflammatory bone diseases (Sloan et al. 2013).

It has been critically discussed whether ex vivo bone cultures requiring specific and/or intact bones can significantly reduce the amount of experimental animals used, when per animal often only two conditions can be tested (Barrach and Neubert 1980; Lessmollmann et al. 1976). Better efficiency in reducing the number of experimental animals is given when the explanted bones are sliced, *e.g.,* in the model described by Srinivasaiah et al. where femurs of young rats are sliced into discs with a thickness of each 300 µm (Srinivasaiah et al. 2019). Similar holds for mandible slice cultures. Unfortunately, destroying the intact explanted organ may affect the cellular composition within the model, as certain cell types can only be found in a specific niche within the organ (Birbrair and Frenette 2016; Morrison and Scadden 2014; Pinho and Frenette 2019). This might be one reason why slice cultures make up the least proportion of ex vivo bone growth studies (Abubakar et al. 2016). So far, mandible or molar slice cultures are preferably used to investigate stem cell behavior and bone repair (Alfaqeeh and Tucker 2013; Colombo et al. 2015; Marino et al. 2016; Smith et al. 2010). The main advantage of mandibular slice cultures is that they can be transferred into the human situation. To do so, immature molar slices from young adults were cultured for several days, to investigate biocompatibility of filling materials and to investigate odontoblast response to damage, eliminating possible species-dependent differences (Melin et al. 2000; Tecles et al. 2008).

Similarly, trabecular core cultures utilize bones of larger animals and even humans (Davies et al. 2006; Endres et al. 2009; Kluter et al. 2020; Knothe Tate and Knothe 2000; Rawlinson et al. 1991; Simpson et al. 2009; Stoddart et al. 2006; Templeton et al. 2015; Vivanco et al. 2013). The larger trabecular core cultures are used to investigate bone metabolism, especially in response to mechanical load (David et al. 2008; Davies et al. 2006; Knothe Tate and Knothe 2000), but the model can also be used to investigate biocompatibility of materials during defect healing (Kluter et al. 2020), or as model to investigate cancer-dependent effects on bone (Salih 2019). In contrast to slice cultures, which are comprised of a trabecular and cortical bone compartment, trabecular core cultures mainly consist of trabecular bone. Therefore, trabecular core cultures represent a great model to investigate alterations in bone metabolism and biocompatibility of materials. The possibility to generate these ex vivo cultures from human bone slices or biopsies further allows investigation of molecular mechanisms in metabolic bone diseases, when the bone samples are obtained from patients with the underlying disease (Bellido and Delgado-Calle 2020; Owen and Reilly 2018). When the models should be used for screening approaches, one has to keep in mind the metabolism of the different drugs within the body, *e.g.,* first path effects and metabolism in the liver. When screening for effects of established drugs on bone metabolism, this may not be a problem, as the known metabolites can be tested. It becomes challenging, when novel substances shall be screened–in this case, only direct effects of the drugs on the bone tissue can be observed, *e.g.,* seen with implant coatings. Furthermore, the availability of the required native human or large animal material is still limited, such that these models cannot be easily used for large-scale drug/substance screenings. This raises the need for a permanently available and up-scalable in vitro model, which represents metabolism and vasculature of human bone.

### In vitro models

In the past years, several attempts have been explored to establish model systems that represent bone metabolism and vasculature. It is self-explanatory that these processes cannot be displayed in conventional 2D mono-cultures. In the conventional 2D cultures on cell culture plastic, the organic (mostly collagen) and inorganic (mostly hydroxyapatite) matrix characteristics for bone are missing. However, this bone matrix essentially functions as regulators for bone cell function and differentiation (Florencio-Silva et al. 2015; Green et al. 1995). Furthermore, in most cases, a co-culture of 2 or sometimes 3 cell times was described, which cannot adequately represent the in vivo situation. Being able to mix only a limited amount of cell types with each other, the purpose of the in vitro models has to be clearly defined. When investigating the biocompatibility of implant materials or bone metabolism, bone forming osteogenic cells and bone resorbing osteoclastic cells are essential. It has been reported that vessel forming endothelial cells or even hematopoietic cells affect bone metabolism (Fuchs et al. 2009, 2007); therefore, including these cell types in the co-culture shall be considered. In case of investigating effects during fracture healing, not only addition of hematopoietic and endothelial cells but all sorts of immune cells should be considered, as they comprise the fracture hematoma in vivo (Kolar et al. 2010). Hematopoietic cells and immune cells are also crucial for testing biocompatibility of materials, but in this case, the so-called hemocompatibility and inflammatory response are investigated separately.

Previous attempts to establish co-cultures based on primary human osteoclasts, osteoblasts, and/or osteocytes to resemble bone metabolism proved to be strongly donor-dependent and time-consuming:

- To obtain a number of osteoblasts and/or osteocytes sufficient for experiments, the cells have to be cultured for several weeks up to months;
- The use of osteoprogenitor cells, *e.g.,* MSCs derived from bone marrow or fat tissue may speed up the expansion time, but might elongate the period of differentiation (Ehnert et al. 2011; Zachos et al. 2014);
- By that time, the donors will no longer be available for a blood donation to generate osteoclasts; therefore, a compatible donor for the isolation of monocytes has to be found;
- Meeting these requirements, the co-culture itself lasts up to 6 weeks depending on the protocol used (Forte et al. 2016; Greiner et al. 2009; Heinemann et al. 2011, 2013; Jablonski et al. 2016; Papadimitropoulos et al. 2011; Penolazzi et al. 2016; Tortelli et al. 2009; Wu et al. 2015; Zehnder et al. 2017);

To quickly obtain larger amounts of cells, a few models utilize human cell lines for their co-cultures (Jablonski et al. 2016; Wu et al. 2015). This has the advantage that large amounts of cells can be obtained in a short time to provide sufficient amount of model material for larger screening approaches. However, one cannot neglect that the choice of the cell line has to be carefully done to represent best the primary counterpart and even then the use of cell lines cannot represent the inter-individual differences characteristic for humans.

**Fig. 3:**
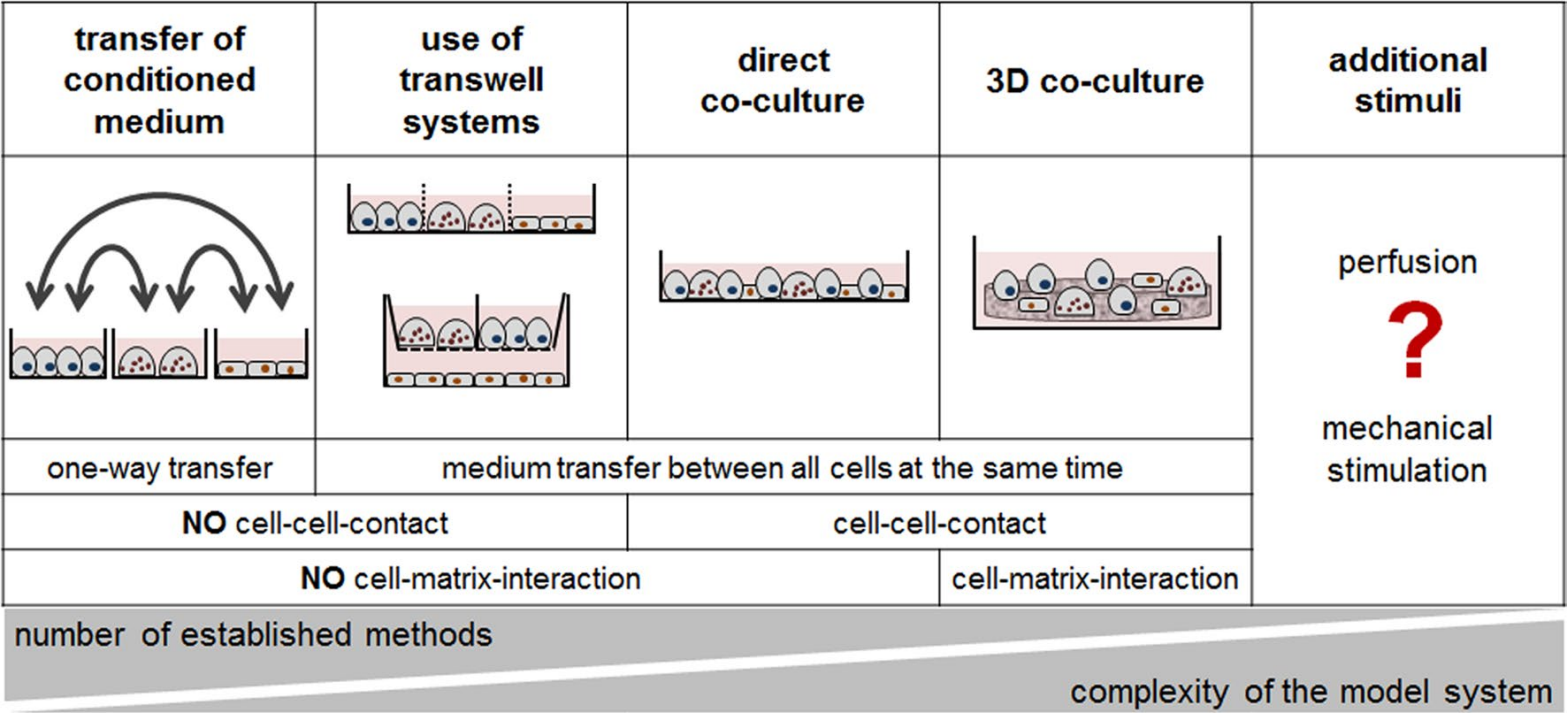
Advantages and disadvantages of the different available *in vitro* bone models. With increasing complexity of the model system, fewer methods are available for analyses

The co-culture models not only differ in the type of cells used. There are also essential differences in the individual setup of the co-cultures (Fig. 3). Again considering only co-cultures of bone forming and resorbing cells:

- Not all co-cultures allow direct cell–cell interactions (Forte et al. 2016);
- Even less provide cell–matrix interactions (Forte et al. 2016; Heinemann et al. 2011, 2013; Tortelli et al. 2009; Wu et al. 2015);
- Only a few models respect the natural 3D organization of cells (Heinemann et al. 2013; Papadimitropoulos et al. 2011; Penolazzi et al. 2016; Tortelli et al. 2009);
- And only one model considers mechanical stimulation of bone cells (Penolazzi et al. 2016).

Recently, there is growing evidence that osteogenic and osteoclastic cells are not only influenced by the surrounding matrix (Florencio-Silva et al. 2015; Green et al. 1995) and mechanical stimuli (Frost 1994), but also strongly by bone marrow resident adipocytes (Horowitz et al. 2017) or endothelial cells of blood vessels (Kirkpatrick et al. 2011). However, it is not yet known how changes in bone metabolism affect vascularization or vice versa how an altered vasculature affects bone metabolism. So far, most co-culture models focused on the influence of osteogenic cells and endothelial cells in direct and indirect co-cultures (Fuchs et al. 2007; Ghanaati et al. 2011; Hofmann et al. 2008; Li et al. 2014; Ma et al. 2020; Shi et al. 2016; Sun et al. 2016), not considering, that the interplay of the presence of osteoclastic cells may alter the function of the osteogenic cells or vice versa (Zachos et al. 2014). Even more complex is the situation, when investigating fracture healing. There, the fracture hematoma consisting of hematopoietic cells and different immune cells is thought to be the key driver for fracture healing (Kolar et al. 2010). Pfeiffenberger et al. have described an equine in vitro model, addressing this complex situation, using coagulated blood as 3D carrier for mesenchymal stem cells (Pfeiffenberger et al. 2019).

Furthermore, the bone is not an isolated organ. Many drugs get after first path effect activated and metabolized in the liver. Therefore, the discussion arose whether bone cultures shall be included in the so-called organ on a chip approaches. A simpler way would be to test the effect of not only native but also activated or metabolized substances on the bone cultures. As elaborated above this may not be a problem when screening for effects of established drugs on bone metabolism but becomes challenging, when novel substances shall be screened. However, this approach is only feasible when a suitable human ex vivo or in vitro model is available in large quantities.

### Challenges of the in vitro models

**Fig. 4:**
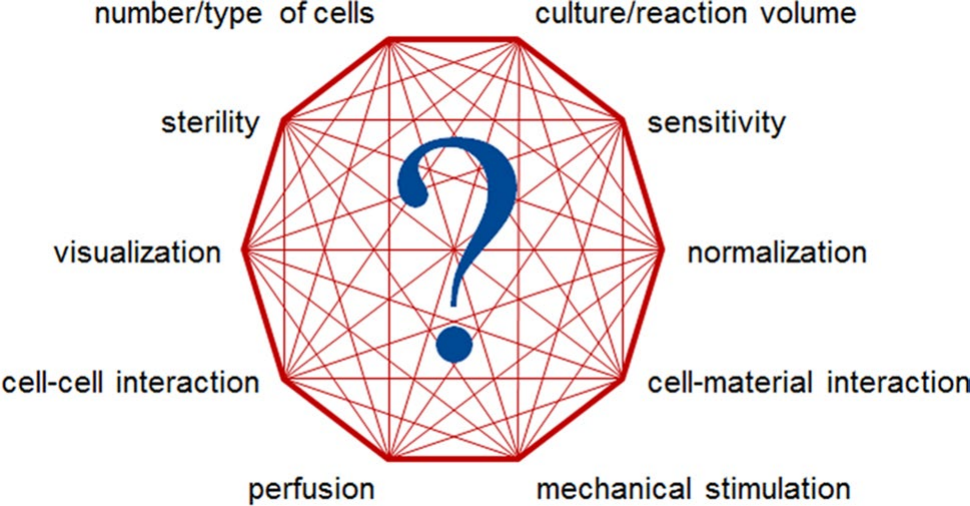
Schematic overview of the factors influencing each other in complex model systems

Increasing the number of cell types in a co-culture while addressing the requirements on 3D conformation, matrix composition, and mechanical stimuli, *e.g.,* mechanical load or perfusion, significantly increases the complexity of the culture model, which, in turn, limits the availability of methods for analysis (James Kirkpatrick et al. 2007). For overview see Fig. 4.

### Medium composition

All cell types have individual requirements on the medium composition. Therefore, considering different cell types for a co-culture may require modulation of the medium composition. For example, osteogenic differentiation medium frequently contains dexamethasone, which is a well-known immune-suppressant. By altering the immune response of mononuclear cells, the glucocorticoid may interfere with osteoclast formation in co-cultures addressing bone metabolism (Kim et al. 2006; Warabi et al. 2001). Dexamethasone in the differentiation medium may be replaced by cholecalciferol (vitamin D_3_), which acts via the vitamin D receptor both on osteogenic and osteoclastic cells (Ehnert et al. 2011). And yet mononuclear cells as precursors for osteoclasts need to be activated to attach to the cell culture plastic and differentiate into osteoclasts. Human myeloid cell lines, *e.g.*, THP-1 or HL-60, normally get activated upon exposure to PMA (phorbol 12-myristate 13-acetate) (Daigneault et al. 2010; Padilla et al. 2000), which, in turn, may affect function of other cells in the co-culture. In this case, sequential seeding of the cells may be inevitable to prevent an unwanted exposure to PMA. Addition of PMA is not required when using murine macrophage cell lines, *e.g.,* RAW 264.7 or J774, which are adherent cells by nature. Osteoclast differentiation is normally induced by the addition of macrophage colony-stimulating factor (M-CSF) and receptor activator of NF-κB ligand (RANKL) to the medium. These two factors are produced by many osteogenic cells, such that these supplements may be reduced or even removed from the co-culture medium. When additionally, vascularization shall be addressed, endothelial cells have to be included in the co-culture model. Endothelial cells normally grow in more complex media than osteogenic or osteoclastic cells. The various growth factors included in their medium, *e.g.,* vascular endothelial growth factor 165, epidermal growth factor, basic fibroblast growth factor, or insulin-like growth factor, may affect the other cells in the co-culture, possibly aggravating the inclusion of these cells in a co-culture.

### Choice of cell types and cell–cell ratios

Even simpler seem the questions for the adequate cell types and cell numbers for a co-culture. Resuming the example of M-CSF and RANKL, which should be produced by osteogenic cells–however, when comparing the three most commonly used osteogenic cell lines MG-63, Cal-72, and SaOS-2, expression of *M*-*CSF* seems to increase with increasing degree of osteogenic differentiation of these cells (Rochet et al. 2003). However, depending on the subset of cells in culture, *M*-*CSF* expression may be even absent (Trojani et al. 2005). We have observed similar effects with *RANKL* expression: while SaOS-2 cells express *RANKL* in levels comparable to human osteoblasts, Cal-72 cells barely express *RANKL*. These cell lines not only differ in their expression profile, but also in proliferation and osteogenic differentiation (Lauvrak et al. 2013)—therefore, when considering the highly proliferative MG-63 cells for co-culture, less cells might be needed than when using Cal-72 cells or SaOS-2 cells. Even more difficult is finding the right density of myeloid cells applied to the co-culture. During seeding, the density of the myeloid cells should be high to allow fusion of the cells to pre-osteoclasts (Jansen et al. 2012). However, to resemble the cell–cell ratio observed in bone, during maturation, the cell ratio of osteoclasts and osteogenic cells has to shift strongly towards the osteoblasts and osteocytes, which make up to 95% of the cells in the bone (Florencio-Silva et al. 2015). Similar holds for the endothelial cells. These cells normally require a certain cell density on soft surfaces to migrate towards each other, align, and form tubes (Arnaoutova and Kleinman 2010). So far, it is not known how these cells may act in a direct co-culture, when the matrix gets mineralized by osteoblasts or even degraded by osteoclasts.

### Characteristics of the carrier material

Taking into consideration that the surrounding matrix is not only essential for tube formation of endothelial cells but also regulates bone metabolism, the right choice of material seems to be fundamental. While endothelial tube formation assay is mainly done on soft hydrogels in 2D culture (Arnaoutova and Kleinman 2010), caving in soft hydrogels may prevent the fusion of monocytic cells early in osteoclastogenesis. Furthermore, there is evidence that 3D arrangement of cells on stiff and porous carriers favors osteogenic maturation of cells (Griffith and Swartz 2006). For bone tissue engineering, a large number of different hydroxyapatite-containing scaffolds exist (Ayobian-Markazi et al. 2012; George et al. 2006; Jain et al. 2015; Mayr-Wohlfart et al. 2001), which favor cell attachment, proliferation, and maturation of osteogenic cells (Jain et al. 2015; Thein-Han and Misra 2009). Therefore, 3D cultures seem to be more beneficial for the differentiation when compared to 2D cultures (Bulnheim et al. 2014). Therefore, when a co-culture of osteogenic and osteoclastic cells with endothelial cells is aspired, an indirect co-culture approach, *i.e.,* by separation with permeable culture inserts, could be advantageous as the soft hydrogel required by the endothelial cells can be easily combined with a stiffer and porous 3D carrier required by the bone cells. However, 3D cultures also raise new challenges, *i.e.,* proper adjustment of the physical characteristics of the 3D matrix to the human physiology, uniform cell seeding on scaffolds, and adequate supply with nutrients (Henkel et al. 2013), factors associated with each other. Pore size, porosity, water uptake rate, and stiffness are essential physical characteristics of scaffolds (Haussling et al. 2019; Weng et al. 2020; Zhu et al. 2018). Pore size and porosity affect both cell attachment and cell infiltration into the scaffold (Murphy and O’Brien 2010). Furthermore, these factors and the water uptake rate are directly associated with nutrient and waste diffusion, a factor that may be actively influenced by applying medium flow to the cultures (Murphy and O’Brien 2010). There are studies, showing that carriers with stiffness over 60 kPa favor osteogenic differentiation of MSCs (Engler et al. 2006; Sun et al. 2018). Lowering the stiffness of scaffolds may induce expression of stem cell markers, *e.g.,* Sox2, in MSCs, which proved to inhibit osteogenic differentiation (Ding et al. 2012; Marcellini et al. 2012; Park et al. 2012; Seo et al. 2013) in favor for adipogenic differentiation (Zhao et al. 2014). However, the chosen scaffolds should also not be too stiff to pass on mechanical stimuli to cells (Dawson and Oreffo 2008).

### Reaction volume of the model system

Besides the effects, the physical characteristics of the carrier material might have on the co-culture; the ratio of culture medium to cell number has to be considered, too. In a static 3D co-culture to completely cover cells on porous scaffolds with medium, the volume is likely to be increased. In this case, the increase in medium volume may be at least partly be compensated by the increase in surface area available for the cells to attach and grow (Hadida and Marchat 2020; Zhang et al. 2018). However, applying mechanical forces or perfusion to a model system would increase the required culture/reaction volume even more without any compensation in cell numbers and may increase the risk for contaminations due to the increased number of structural junctions (Hadida and Marchat 2020). An increase in culture/reaction volume would dilute factors secreted by cells in the culture medium and thus effectively lowers the sensitivity of established analytical methods. However, the use of scaffolds bares also other challenges:

- The scaffold limits microscopic analyses—the use of fluorophores may circumvent some of these limitations (Tendulkar et al. 2016); however, penetration depth and possible autofluorescence of the material remain limiting factors.
- Assay substrates and reaction products require time for perfusion.
- Assay substrates or reaction products may react with the scaffold material.
- The scaffold material or particles released from the carrier into the reaction solution may disturb photometric measurements.
- Cells may not be efficiently released from the scaffolds, which may disturb or even impede preparation of RNA or protein lysates for analyses.

## Normalization of functional assays in complex in vitro models

Undoubtedly, co-cultures represent a great chance for research and screening purposes; however, the described mainly technical limitations suppress their full potential. While some limitations can be addressed by a deliberate choice of cells and materials, others are not that easy to address. Often forgotten is the cell-specific normalized of functional measurements in co-cultures, an important issue that will be further explored in the following section. For overview, see Table 1.

**Table 1:** Suitability of different normalization methods for complex culture systems

	Mitochondrial activity						Stable transfection (*e.g.* GFP) of cell lines	Cellular stainings									Total protein content		Total DNA content		
	ATP	NADH/NADPH	MTT	XTT, MTS, WST-1	Resazurin (photometric)	Resazurin (fluorescent)		DoO, DiI, DiD, DiR + derivatives	FM Dyes + derivatives	Cell Tracker (*e.g.* CFSE)	Calcein – AM + derivatives	DNA dyes (*e.g.* DAPI or SYTOX)	DNA dyes (*e.g.* Hoechst 33342)	Histological stains (*e.g.* H&E)	Immunohistological staingings	Immunofluorescent stainings	SRB staining	Detection of AA (*e.g.* Lowry, BCA)	Quantification of DNA (photometric)	Quantification of DNA (fluorescent)	Quantification of DNA (qPCR)
Detects viable cells	**+**	**+**	**+**	**+**	**+**	**+**	**(+)**	**−**	**−**	**−**	**+**	**−**	**−**	**−**	**−**	**−**	**−**	**−**	**−**	**−**	**−**
Detects all cells in the system	**−**	**−**	**−**	**−**	**−**	**−**	**−**	**+**	**+**	+*	**−**	**+**	**+**	**+**	**+**	**+**	**+**	**+**	**+**	**+**	**+**
Time-resolved measurements possible	**−**	**−**	**−**	**−**	**−**	**−**	**+**	**+**	**+**	**+**	**−**	**−**	**−**	**−**	**−**	**−**	**−**	**−**	**−**	**−**	**−**
Method toxic to the cells	**+**	**−**	**+**	**−**	**−**	**−**	−*	**−**	**−**	**−**	**−**	*na*	**−**	*na*	*na*	*na*	*na*	*na*	*na*	*na*	*na*
Method affects cell functions	*na*	**−**	*na*	**−**	**−**	**−**	−/+	−/+	−/+	−/+	**−**	*na*	**+**	*na*	*na*	*na*	*na*	*na*	*na*	*na*	*na*
Sensitive towards pH changes	−/+	**−**	−/+	−/+	**+**	**+**	**−**	**?**	**?**	**−**	**+**	*na*	**+**	*na*	*na*	*na*	*na*	*na*	*na*	*na*	*na*
Affected by cellular stress	−/+	−/+	**+**	**+**	**+**	**+**	**?**	**−**	**−**	**?**	−/+	*na*	**−**	*na*	*na*	*na*	*na*	*na*	*na*	*na*	*na*
End-point measurement	**+**	**+**	**+**	**+**	**+**	**+**	−/+	−/+	−/+	−/+	**+**	**+**	**+**	**+**	**+**	**+**	**+**	**+**	**+**	**+**	**+**
Detection method	**L**	**F**	**A**	**A**	**A**	**F**	**F**	**F**	**F**	**F**	**F**	**F**	**F**	**A**	**A**	**F**	**A**	**A**	**A**	**F**	**F**
Requires co-factors	**−**	**−**	**+**	**+**	**+**	**+**	+*	**−**	**−**	**−**	**−**	**−**	**−**	**+**	**+**	**+**	**−**	**+**	**−**	**+**	**+**
Treatment of cells required (*e.g.,* fixation, permeabilization, lysis, etc.)	**−**	**−**	**−**	**−**	**−**	**−**	**−**	**−**	**−**	**−**	**−**	**+**	**−**	**+**	**+**	**+**	**+**	**+**	**+**	**+**	**+**
Quantification requires additional step (*e.g.,* resolving, qPCR, imaging, etc.)	**−**	**−**	**+**	**−**	**−**	**−**	**+**	**+**	**+**	**+**	**+**	**(+)**	**(+)**	**+**	**+**	**+**	**+**	**+**	**−**	**+**	**+**
Sensitivity of the signal	**G**	**M**	**W**	**W**	**W**	**G**	**M**	**M**	**M**	**G**	**G**	**G**	**G**	**M**	G*	G*	**W**	G*	**W**	G*	**G**
Stability of the signal	**W**	**W**	**G**	**G**	**G**	**M**	**M**	**M**	**M**	**M**	W*	**M**	**M**	**G**	**G**	**W**	**G**	**G**	**G**	M*	**M**
Quantification	**M**	**M**	G*	G*	G*	G*	**W**	**W**	**W**	G*	G*	G*	G*	**W**	**W**	**W**	**M**	**G**	**M**	**G**	**G**
Modifications required for 2D-3D transfer	**G**	**G**	**W**	**W**	**W**	**W**	**M**	**M**	**M**	**M**	**M**	**M**	**M**	**M**	**M**	**M**	*na*	*na*	**M**	**M**	**M**
Interference with 3D matrices	**+**	**+**	+*	+*	+*	+*	+*	+*	+*	+*	+*	+*	+*	+*	−*	−*	**+**	**+**	+*	−*	−*
Influenced by altered volumes	**−**	**−**	**+**	**+**	**+**	**−**	−*	−*	−*	−*	**+**	**−**	+*	**−**	**−**	**−**	**−**	**+**	**−**	**−**	**−**
Uniform between different cell types	**−**	**−**	**−**	**−**	**−**	**−**	**+**	**+**	**+**	**+**	**−**	**+°**	**+°**	−/+	−/+	−/+	−/+	−/+	**+°**	**+°**	**−**
Can differentiate between cell types	**−**	**−**	**−**	**−**	**−**	**−**	+*	+*	+*	+*	**−**	**−**	−/+	+*	+*	+*	**−**	**−**	**−**	**−**	+*
Can be passed to other cell in the system	*na*	*na*	*na*	*na*	*na*	*na*	**+**	**+**	**+**	**+**	*na*	*na*	**−**	*na*	*na*	*na*	*na*	*na*	*na*	*na*	*na*

*A* adsorption, *AA* amino acids, *F* (auto)fluorescence, *G* good/high, *L* luminescence, *M* medium; *na* not applicable, *W* weak/low

* Depending on the application; ° exception is multinucleated cells

### Mitochondrial activity for normalization

Most publications use the mitochondrial activity of cells for viability measures and normalization. There exist a considerable amount of assays to quickly and simply determine the bioenergetic status of intact cells (Brand and Nicholls 2011). Normalizing a system to the mitochondrial activity has the beauty that theoretically only viable cells are taken into consideration.

In cellular monolayers, often the bioenergetic intermediates ATP, NADH, or NADPH are determined. Though it is appealing to measure the amount of ATP in a cell using luminescence, the amount of cellular ATP does not safely report mitochondrial function as most of the adenine nucleotide in cells is present as ATP (Brand and Nicholls 2011). Therefore, many researchers determine the cellular NADH or NADPH. This is executed either by measuring autofluorescence or using colorimetric or fluorescent substrates which get reduced in an NADH- or NADPH-dependent manner (Henriques et al. 2011).

Examples for colorimetric assays are the classical MTT (3-(4,5-dimethylthiazol-2-yl)-2,5-diphenyl-2H-tetrazolium bromide) assay, or assays using water-soluble tetrazolium derivatives *e.g.* XTT (2,3-bis(2-methoxy-4-nitro-5-sulfophenyl)-2H-tetrazolium-5-carboxanilide inner salt), MTS (3-(4,5-dimethyl-2-thiazolyl)-5-(3-carboxymethoxy-phenyl)-2-(4-sulfophenyl)-2H-tetrazolium inner salt), or WST-1 (2-(4-iodophenyl)-3-(4-nitrophenyl)-5-(2,4-disulfophenyl)-2H-tetrazolium monosodium salt). While MTT is reduced to insoluble formazan crystals, the second-generation tetrazolium salts XTT, MTS, and WST-1 are reduced to hydrosoluble colored formazans in mitochondria of living cells, which ultimately reduces the toxicity of the assays and abolishes the need for an additional step of solubilization (Henriques et al. 2011). However, the water-soluble tetrazolium methods require an intermediate electron transfer reagent, mainly PMS (5-methyl-phenazinium methyl sulfate) or menadione, which represents the reduced agent in the assay transferring its electrons to the tetrazolium salts. Noteworthy, the production of colored aqueous formazan is amplified by the use of PMS in the assays, thereby increasing their detection limit (Henriques et al. 2011).

The non-toxic Resazurin conversion assay can be measured either photometric or fluorescent, which increases the sensitivity of the assay significantly. The water-soluble Resazurin (blue and non-fluorescent) is reduced to highly fluorescent Resorufin (pink) in mitochondria of intact cells. This reaction requires also NADH or NADPH as co-factor. Therefore, the amount of produced Resorufin is supposed to linearly increase with the amount of cellular NADH and NAPDH content available (Brand and Nicholls 2011).

Water-soluble assays have the advantage that the formed product is actively exported into the culture supernatant, which allows measurements in real time. One has to consider that the substrates and products may require time to perfuse in 3D cultures and may even react with the scaffold material. Furthermore, the scaffold material itself may limit the use of photometric assay–reaction solution must be transferred to separate plates or cuvettes for measurement, which still bears the risk that scaffold particles resolved in the reaction solution may interfere with the optical measurement. Measuring fluorescence, *e.g.,* in the Resazurin conversion assay, is less susceptible for such interference.

**Fig. 5:**
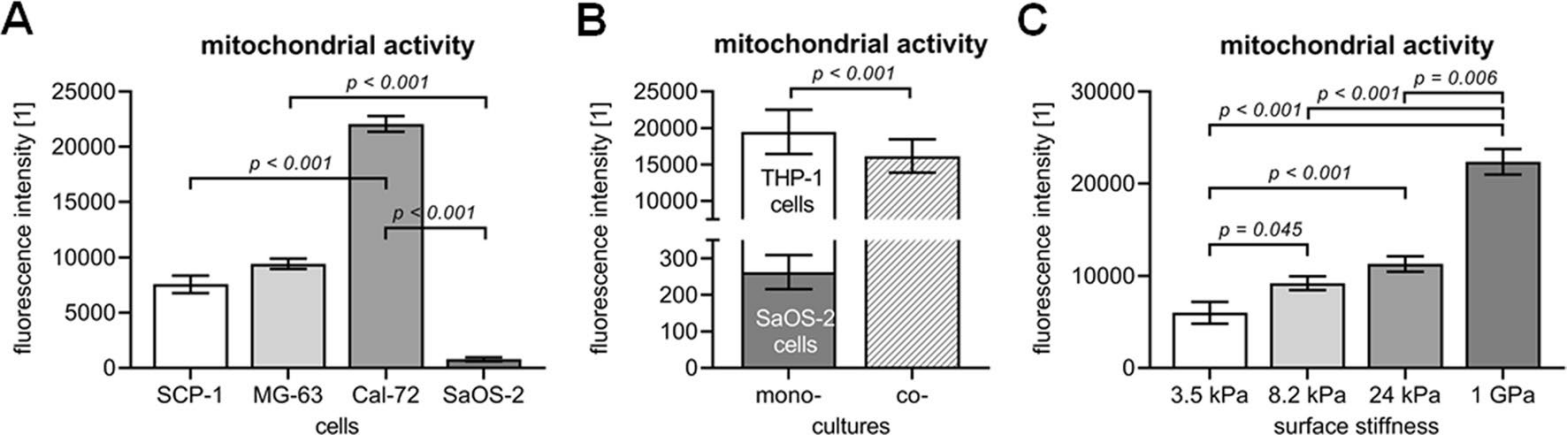
Limitations of using mitochondrial activity for normalization. Mitochondrial activity was assessed by Resazurin conversion assay. **a** Mitochondrial activity varies between different osteogenic cell lines. Exemplary, 5 * 10^4^ cells of the SCP-1, MG-63, Cal-72, and SaOS-2 cell line were seeded, and after 24 h, mitochondrial activity was determined. **b** Mitochondrial activity was measured in THP-1 and SaOS-2 cell cultures both in mono-culture and direct co-culture. **c** Surface stiffness affects mitochondrial activity. 5 * 10^4^ cells of the same cell line were seeded on surfaces with different stiffness, and after 24 h, mitochondrial activities were measured. Experiments were repeated three times (N = 3) in triplicates (n = 3). Comparison of groups was performed by Kruskal–Wallis test followed Dunn’s multiple comparison test

The described assays may also be used to measure viability in ex vivo bone cultures. However, these methods have been originally developed for the conventional 2D mono-cultures. Using them in more complex 3D systems, e.g., co-cultures or ex vivo cultures, often requires adaption of the methodology. For that reason, there are certain limitations of the assays which the researchers have to be aware. 3D carriers or their solutes may interfere with the optical measurements, especially, when the assay is based on adsorption measurements. In case of fluorescent assays, possible autofluorescence of the 3D carrier material may disturb the measurement. In most cases, it is sufficient to simply transfer the reaction solution to a new microwell plate to eliminate the disturbances. When transferring the reaction solution is required, kinetic measures cannot be easily done any more. Even more challenging is the fact that the assays cannot differentiate between different cell types. Cell lines, even from the same lineage, do not have the same mitochondrial activity (Fig. 5a). This is not a limitation when mono-cultures are analyzed. In case of co-cultures, however, the increase in Resorufin conversion cannot be traced back to the different cell types. This is exemplarily shown in Fig. 5b, where the mitochondrial activity in the mono-cultures is in sum higher than the mitochondrial activity in the corresponding co-culture. It becomes even more complex, when cells are cultured on 3D carriers, as the mitochondrial activity of the same type of cell may change in response to stress such as slight variations in the pH or the stiffness of the carrier (Fig. 5c). In culture systems aiming to investigate bone function, where formation and degradation of matrix are a functional readout, this represents another limiting factor. From toxicological assays, it can be observed that cells under stress often show increase mitochondrial activity, prior to measurable damage by leakage of lactate dehydrogenase or reduction in total protein or DNA. Normalizing the mitochondrial activity to the total protein content may even be used as measure for cellular stress. Therefore, when using mitochondrial activity for normalization, it is advisable to use also alternative measures, *e.g.,* total protein or DNA content, or glucose consumption (Haussling et al. 2019), although these measures also cannot easily distinguish between different cell types in a co-culture.

### Optical cell tracking for normalization

Some studies also use different fluorescent labels to discriminate between different cell types in a co-culture. The available trackers and associated methods allow a great variety in the experimental setup. Most easily applied are the so-called cell trackers. Long-chain carbocyanines, *e.g.* DiO (3,3′-dioctadecyloxacarbocyanine perchlorate–green), DiI (1,1′-dioctadecyl-3,3,3′,3′-tetramethylindocarbocyanine perchlorate–yellow), DiD (1,1′-dioctadecyl-3,3,3′,3′-tetra-methylindodicarbocyanine perchlorate–red), DiR (1,1′-dioctadecyl-3,3,3′,3′-tetramethylindotricarbocyanine iodide–far red), or their derivatives, are weakly fluorescent in aqueous solution but highly fluorescent and even quite photostable when incorporated into cell membranes (Texier et al. 2009). These lipophilic dyes diffuse laterally within the plasma membrane of cells in culture medium or other aqueous buffers, resulting in staining of the entire cell. Such cell tracers have to be used with care-extensive washing of cells which is recommended to avoid the so-called micro-environmental contaminations, which occurs by passing on the dye from one cell to the neighboring cell (Lassailly et al. 2010). In co-cultures including osteoclast, this might not be sufficient, as osteoclasts are derived from activated myeloid cells, which possess phagocytic activity and thus may actively spread the tracers (Lassailly et al. 2010).

Comparably, lipophilic styryl dyes, also referred to as FM dyes, diffuse rapidly but also reversibly into plasma membranes of cells resulting in a strong fluorescent enhancement (Wu et al. 2009). FM dyes, *e.g.,* FM1-43 (N-(3-triethylammonium-propyl)-4-(4-(dibutylamino)styryl)pyridinium dibromide–red), FM2-10 (N-(3-triethylammonium-propyl)-4-(4-(diethyl-amino)styryl)pyridinium dibromide–orange), FM4-64 (N-(3-triethylammonium-propyl)-4-(6-(4-(diethyl-amino)phenyl)hexatrienyl)pyridinium dibromide–red), or FM5-95 (N-(3-trimethylammoniumpropyl)-4-(6-(4-(diethyl-amino)phenyl)hexatrienyl)pyridinium dibromide–red), are widely used to study endocytosis, vesicle trafficking, or organelle organization in living cells (Bolte et al. 2004). Nowadays, modified dyes which allow fixation of the stain (FX modifications) are available. Furthermore, fine chemical modifications of FM1-43, which is one of the most widely used FM probes, resulted in new probes, SP-468 (red) and SQ-535 (far red), which have enhanced photophysical properties, *e.g.,* reduced crosstalk, higher brightness, or improved photostability (Collot et al. 2019). Similar to the long-chain carbocyanine dyes, it cannot be granted that dyes incorporated into the plasma membranes will not cause micro-environmental background by passing on the stain to neighboring cells or due to phagocytosis.

Live cell dyes work slightly different. The non- or low fluorescent dyes freely pass through plasma membranes into the cells, where they are transformed into cell membrane-impermeable fluorescent reaction products. The best-known representative of this group is calcein-AM (AM = acetoxymethyl) which is part of many live–dead staining kits. The non-fluorescent calcein-AM easily passes the plasma membrane, where it is converted to green-fluorescent calcein by intracellular esterases (Bratosin et al. 2005). The beauty to mark viable cells using fluorogenic compounds, *e.g.,* calcein-AM and its derivatives (blue, orange, and red), has increased their frequency of use in cell and molecular biology. However, leakage of the fluorescent calcein or its interactions with exogenous stimulants, *e.g.,* metal ions or electrochemically generated by-products, have been reported (Miles et al. 2015). Therefore, the use of adequate controls for accurate measurements and valid conclusions is inevitable. Considering live cell dyes for normalization, the same limitations than for measuring mitochondrial activity exist. The live cell dyes will not distinguish between the different cell types, and, therefore, need to be combined with other tracers.

**Fig. 6:**
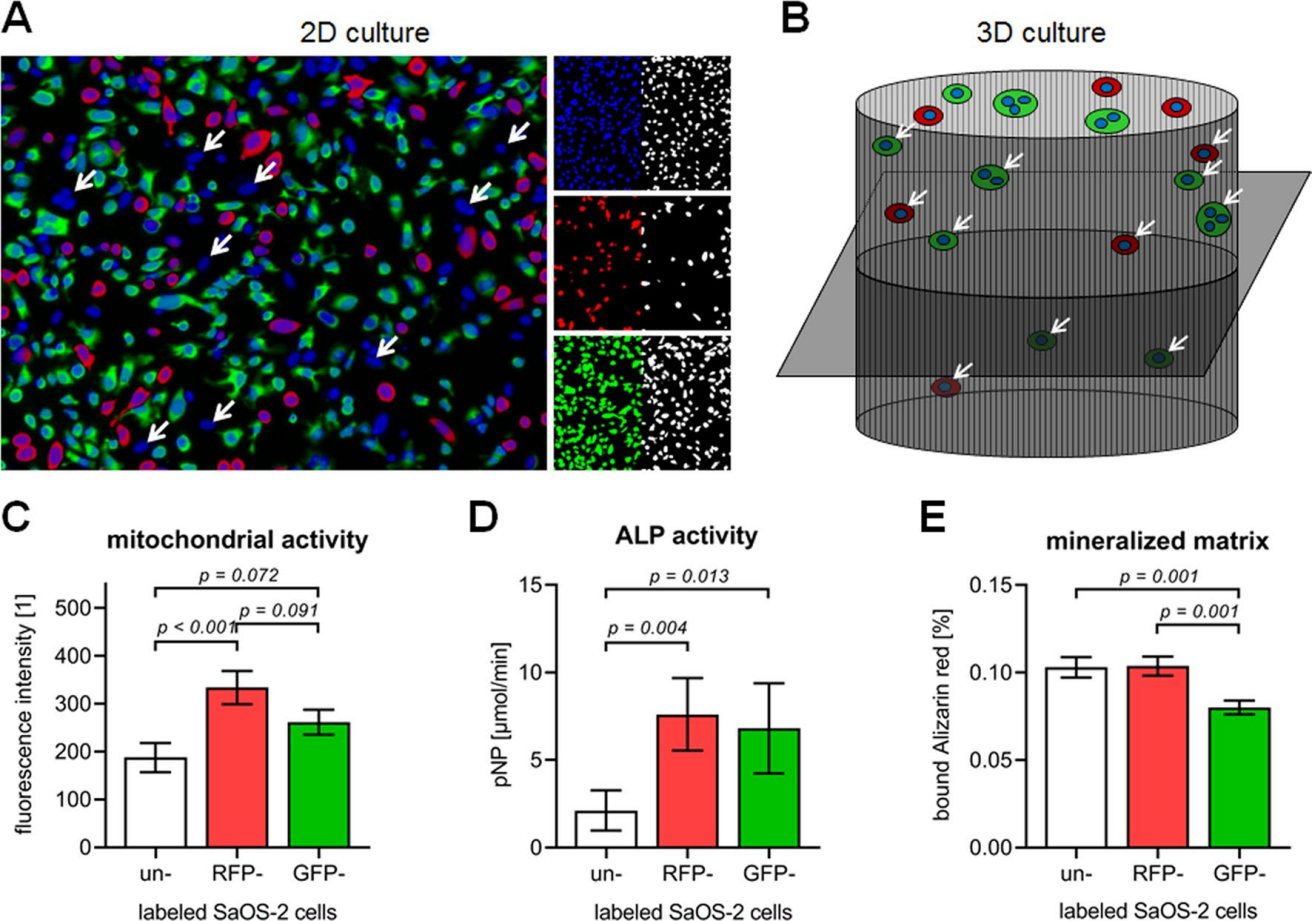
Using fluorescent labels for normalization. SaOS-2 cell line was transfected to over-express a red fluorescent protein (RFP: Addgene plasmid #54642 tdTomato-N1) or a green-fluorescent protein (GFP: Addgene plasmid #54737 sfGFP_N1). **a** RFP-overexpressing SaOS-2 cells (red) were directly co-cultured with THP-1 cells in a ratio of 1:2. After 4 days of conventional 2D culture viable cells were visualized with Calcein-AM (green). Nuclei were counterstained with Hoechst 33342 (blue). **b** Schematic overview on the respective 3D culture. **c** Mitochondrial activity of the un-/labelled SaOS-2 cells on day 4 of culture. **d** ALP activity of the un-/labelled SaOS-2 cells on day 4 of culture. **e** Mineralized matrix formed by the un-/labelled SaOS-2 cells after 10 days of culture. Experiments were repeated three times (N = 3) in triplicates (n = 3). Comparison of groups was performed by Kruskal–Wallis test followed by Dunn’s multiple comparison test

Live cell dyes may be used to visualize living cells also in ex vivo cultures, being aware, that the dyes cannot differentiate between different cell types. In contrast to that, the described lipophilic dyes, which label the cell membranes, would not be of any use for the ex vivo models, unlike invasion of labelled cancer cells should be investigated in a metastasis assay. We have used yet another approach, by permanently labelling different cell types. For a co-culture of SaOS-2 cells and THP-1 cells, we intended to stably transfect (using selection antibiotics) these cell lines with plasmids inducing over-expression of fluorophores. While green and red fluorescent SaOS-2 cell lines could be generated, the transfection efficiency of the myeloid THP-1 cells was not sufficient. Therefore, red fluorescent SaOS-2 cells were co-cultured with THP-1 cells. After exposure to calcein-AM, all living cells should appear green fluorescent, which allowed discrimination between the two cell types. However, some cells only showed the nuclear counterstain (Hoechst 33,342–blue–white arrows) questioning the efficiency of the method (Fig. 6a). Similar to all fluorescent tracers or markers, the method works very well in 2D cultures, but quickly reaches its limits in 3D cultures with non-transparent scaffolds (Fig. 2b) (Tendulkar et al. 2016). To make things worse, our transfected SaOS-2 cells showed altered mitochondrial and alkaline phosphatase (ALP) activity, which in turn affected the formation of mineralized matrix by these cells (Fig. 6c, d). This represent a limitation, one has to keep in mind, when using stably transformed cell lines. These examples show that there is, indeed, a large variety of fluorescent markers and tracers that can be used to track cells in co-cultures. When appropriate controls are done and automated image analysis is possible, these tools represent a very good option to trace and normalize cells in co-cultures, at least in 2D.

### Using DNA for normalization

Another well-established technique to normalize cell cultures is the quantification of total protein or DNA, as this is supposed to be less affected by culture conditions than viability measures. As many cell carriers for 3D cultures contain some protein source, it is not advisable to use total protein content for normalization in 3D settings. It is less likely that cell carriers for 3D cultures contain DNA. Therefore, normalization using DNA may be feasible. Just recently, we have described the DNA-based quantification of different cell types on scaffold-based 3D cultures (Ruoss et al. 2019).

Using this model, which is based on a co-culture of a murine cell line with a human cell line, we could compare different methods to retrieve DNA from cells seeded on the matrix, as well as the feasibility of different DNA quantification methods. Interestingly, the attempt to detach cells from the matrix prior to DNA isolation failed to isolate all DNA that was contained on the matrix. A simple NaOH-based isolation technique (Ehnert et al. 2019b) is frequently used to isolate DNA from tissue samples for genotyping in mice effectively retrieved all DNA from the scaffolds. Comparing different DNA quantification methods showed that the limit of detection (LOD) and consequently limit of quantification (LOQ) were lowest for the fluorescent-based CyQuant assay, which also had the highest sensitivity (101–104%). Surprisingly, neither the LOD and LOQ (~ 1.5-fold higher) nor the sensitivity (95–98%), was significantly higher for the conventional absorption-based quantification of DNA when compared with the CyQuant assay. The highest LOD and LOQ and the lowest sensitivity (88–98%) had the fluorescent-based Hoechst 33342 assay. These three methods can only be used in mono-cultures or to assess the total amount of DNA in a co-culture. To distinguish between the different cell types in the co-culture a PCR using species-specific primers was performed. The PCR-based method showed an LOD, LOQ, and sensitivity (99%) comparable to the quantification of DNA with the CyQuant assay and conventional absorption-based method, but could differentiate between the two cell types in the co-culture.

**Fig. 7:**
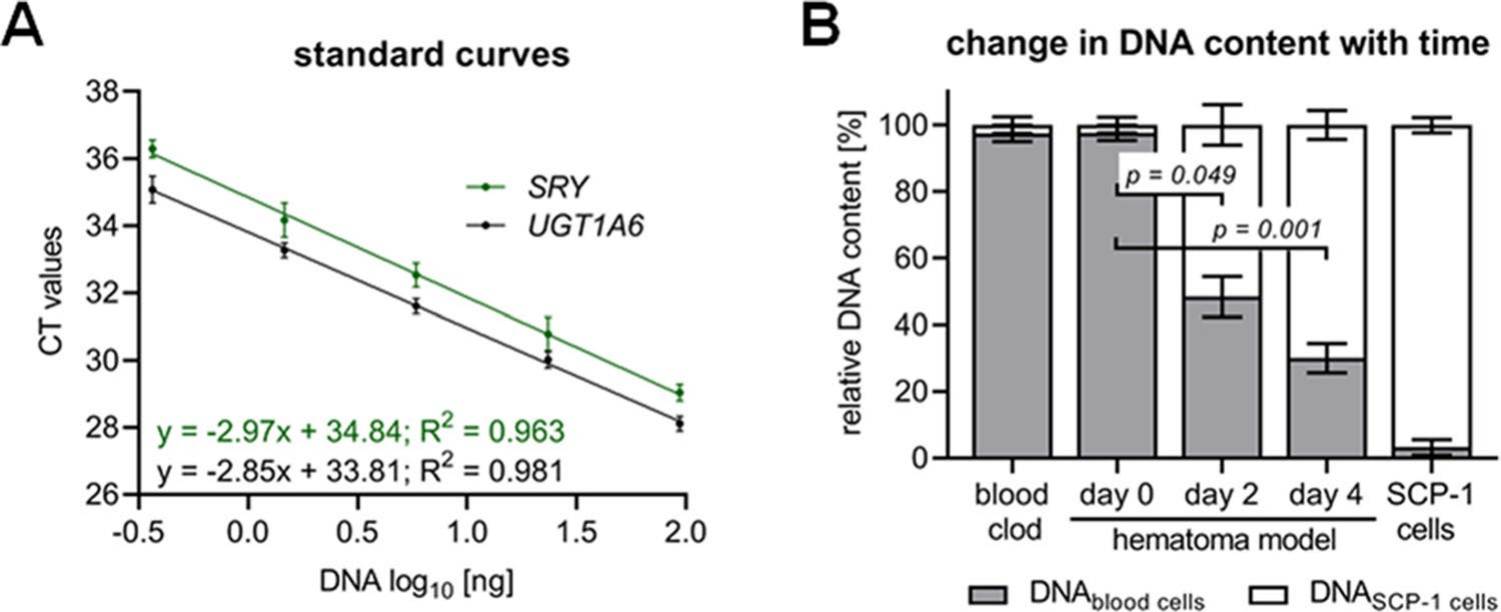
Sex-specific qPCR-based DNA quantification for normalization. An in vitro fracture hematoma was generated as described by Pfeiffenberger et al. (Pfeiffenberger et al. 2019), using blood of a male donor and (female) SCP-1 cells. Normalization of the cells in culture was done by DNA content and sex-specific PCR. **a** Representative standard curves for the sex-specific PCRs. *UGT1A6* located on chromosome 2 is representative for the total DNA amount. *SRY* located on the Y-chromosome is representative for the amount of male DNA. **b** Change in relative DNA amounts in the in vitro fracture hematomas after 2 and 4 days of culture was determined by sex-specific PCR. Experiments were repeated three times (N = 3) in duplicates (n = 2). Comparison of groups was performed by Kruskal–Wallis test followed Dunn’s multiple comparison test

However, aiming for an all human co-culture model, we adapted the PCR-based detection method using sex-specific primers:

- *UGT1A6* (uridine diphosphate glucuronosyl transferase 1A6), located on chromosome 2, was used to determine the total amount of DNA (Ruoss et al. 2019);
- *SRY* (sex-determining region Y), located on the Y-chromosome, was used to determine the amount of male DNA (Drobnič 2006);
- respective standard curves are given in Fig. 7a.

As model system to study early fracture healing an in vitro fracture hematoma was generated as described by Pfeiffenberger et al. (Pfeiffenberger et al. 2019). Briefly, the in vitro fracture hematomas were generated by mixing human whole blood of a male donor and 6 * 10^4^ cells of the immortalized (female) MSC SCP-1 cells. After mixing equal volumes of blood and cells (120 µl) in the presence of calcium ions, in vitro hematomas formed, which remained stable over a culture period of at least 4 days. Whole DNA was isolated after hematoma dissolution and erythrocyte lysis using alkaline DNA extraction with NaOH (Ehnert et al. 2019b; Pfeiffenberger et al. 2019). Comparing the relative DNA amounts determined by *UGT1A6* and *SRY* PCR showed that the relative amount of female DNA (SCP-1 cells) in the model system increased with the culture time.

Despite the possibility that the normalization to DNA content may reach limits, for example when dead cells are entrapped in a 3D environment, the proposed method represents a good alternative to the existing methods when cell-specific normalization of a co-culture system is required. Including a third human cell line may be challenging. A possibility might be the use of immortalized cell lines, e.g., the SCP-1 cell line or the HMEC-1 microvascular endothelial cell line, which offer the possibility to detect (PCR) sequences introduced into the genome during immortalization (hTERT in SCP-1 cells or pSVT in HMEC-1 cells).

## Summary and conclusion

In recent years, there have been great technical developments, when considering co-cultures of bone forming and resorbing cells. The combination of different cell types in 2D and 3D cultures, with and without mechanical stimuli, has been described many times. Advanced 3D carriers and dynamization of the model system allow investigating biocompatibility of implant materials or effect of drugs or their metabolites on bone metabolism. First attempts have been described to also include the interaction with endothelial cells (vascularization) and bone marrow adipocytes in these models, widening their application for investigating systemic bone diseases, *e.g.,* diabetic osteopathy. When it comes to investigating fracture healing in vitro, the situation is even more complex and only first attempt to generate in vitro hematomas have been described. Comparing the huge technical progress with the advances in analytical methods to adequately characterize these models, the required modifications of the techniques from 2D to 3D and from mono-culture to co-culture still lack behind, lowering the full potential of the proposed model systems. Being aware of the strengths and limitations of the different in vitro or ex vivo model systems, they can be used in larger screening approaches, but will not be able to replace in vivo testing for verification.

## Search criteria

On the 5th of April 2020, a search was performed with PubMed and Web of Science, limited to manuscripts in English or German language. The search strategy is summarized in Table 2.

**Table 2:** Search strategy

Search criteria		All	Reviews
#1	“Osteoblast” AND “osteoclast” AND “co-culture”	121	2
#2	“Bone” AND “endothelial” AND “co-culture”	298	13
#3	“Ex vivo” AND “bone metabolism” AND “culture”	19	1
#4	“Ex vivo” AND “bone metabolism” AND “model”	11	0
#5	“Ex vivo” AND “bone growth” AND “culture”	12	0
#6	“Ex vivo” AND “bone growth” AND “model”	21	2
#7	“In vitro” AND “bone” AND “vascularized”	254	14
#8	“Osteoblast” OR “osteoclast” AND “endothelial” AND “in vitro”	297	13
#9	“Osteoblast” OR “osteoclast” AND “endothelial” AND “co-culture”	50	2
#10	#1 OR #2 OR #3 OR #4 OR #5 OR #6 OR #7 OR #8 OR #9	981	43

**Fig. 8:**
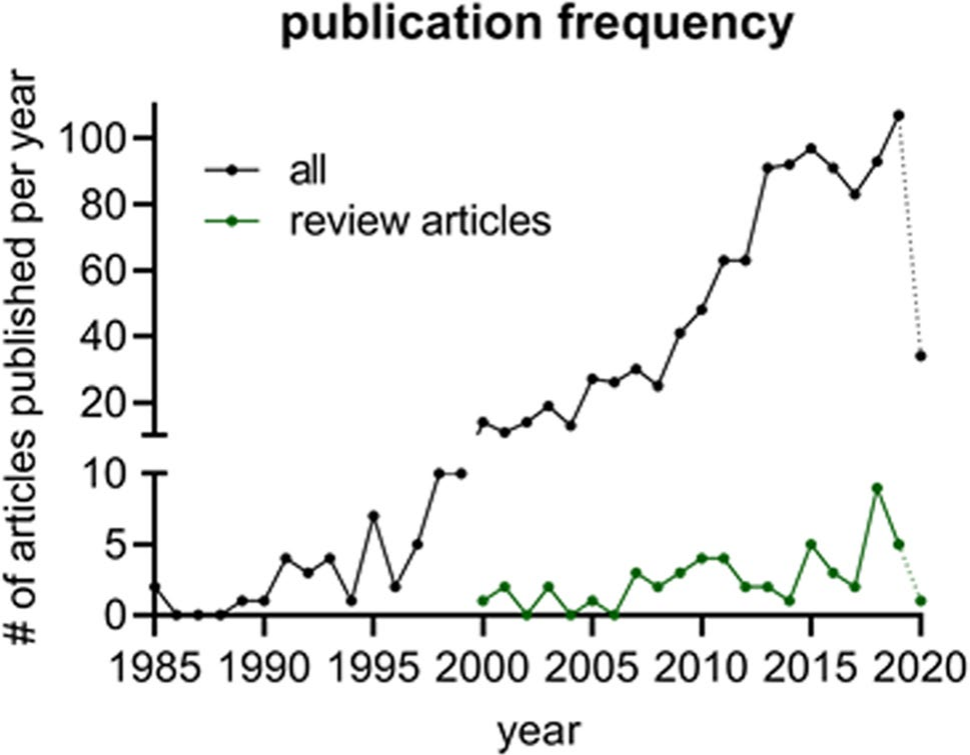
Publication frequency with the above stated search terms (Table 1)

Considering only manuscripts in English or German languages, a total of 981 manuscripts remained for further screening, of which 43 were review articles. In Fig. 8, the number of papers published per year is presented.

## Data Availability

Review article with own data–these can be obtained from the corresponding author upon reasonable request. Not applicable.
